# Measurement invariance of the Kessler Psychological Distress Scale (K10) among children of Chinese rural‐to‐urban migrant workers

**DOI:** 10.1002/brb3.2417

**Published:** 2021-11-13

**Authors:** Qiang Ren, Yong Li, Ding‐Geng Chen

**Affiliations:** ^1^ Department of Sociology Zhejiang University 866 Yuhangtang Rd Hangzhou Zhejiang 310027 China; ^2^ Department of Social Work California State University Bakersfield 9001 Stockdale Hwy Bakersfield California 93311 USA; ^3^ College of Health Solutions Arizona State University 550 N 3rd St Phoenix Arizona 85004 USA

**Keywords:** China, K10, measurement invariance, migrant children, psychological distress

## Abstract

**Introduction:**

Kessler Psychological Distress Scale (K10) is a 10‐item screening tool designed for nonspecific psychological distress. The current study aims to identify a best‐fitting factor structure of the K10, and to test its cross‐gender measurement invariance based on the structure.

**Methods:**

Using convenience sampling, we included 339 (*n* = 192 for boys and 135 for girls) children of Chinese rural‐to‐urban migrant workers in Hangzhou, China.

**Results:**

Confirmatory factor analysis for ordered‐categorical measures revealed a two‐factor structure as the best‐fitting model, in which five items (hopeless, depressed, effort, severely depressed, and worthless) loaded on depression and the other five items loaded on anxiety (tired, nervous, severely nervous, restless, and severely restless). The model held at different levels of the measurement invariance testing, that is, full measurement invariance was not rejected in our sample, suggesting that gender differences as assessed with K10 reflect true differences. Structural invariance testing showed that girls in our sample showed significantly higher levels of depression and anxiety than boys.

**Conclusion:**

These findings support that the K10 is suitable for gender‐comparative research among children of Chinese migrant workers. Using the K10 as a screening tool among this population should be promoted. Limitations and directions for future research were discussed.

## 1 INTRODUCTION

The Kessler Psychological Distress Scale (K10) is a brief screening instrument to assess nonspecific psychological distress such as depression and anxiety among general populations. Based on studies conducted in the general populations of the United States (Kessler, Barker, et al., 2003), Canada (Cairney et al., 2007), Australia (Slade et al., 2011), New Zealand (Browne et al., 2010), and the Netherlands (Donker et al., 2010), the K10 is widely accepted as a screening tool for discriminating people with a mental disorder from those without a mental disorder. The K10 has also been validated in community samples from various non‐western countries. Examples include Asian countries such as China (Bu et al., 2017), Japan (Sakurai et al., 2011), South Korea (Min & Lee, 2015), and India (Fernandes et al., 2011), as well as African countries such as South Africa (Andersen et al., 2011) and Tanzania (Vissoci et al., 2018). In mainland China, the first validation study on the K10 was conducted among Chinese college students, which suggested K10 had good reliability and validity (Zhou et al., 2008).

The evidence on the factor structure of the K10 is inconclusive. A unidimensional factor structure of the K10 has been reported among community samples of the United States (Kessler et al., 2002), Australia (Sunderland et al., 2012), and the Netherlands (Fassaert et al., 2009). Researchers have also uncovered various two‐factor structures of the K10. First, three studies (Lace et al., 2019; O'Connor et al., 2012; Pereira et al., 2019) revealed a two‐factor structure with six items loading on depression (depressed, severely depressed, worthless, effort, hopeless, and tired) and four items on anxiety (restless, severely restless, nervous, and severely nervous). Second, Sunderland et al.’s study (2012) suggested a different two‐factor structure with four items loading on depression (depressed, severely depressed, worthless, and hopeless) and six items on anxiety (restless, severely restless, nervous, severely nervous, effort, and tired). Third, Bu et al. (2017) found that five items (hopeless, depressed, effort, severely depressed, and worthless) loaded on depression and five items (tired, nervous, severely nervous, restless, and severely restless) loaded on anxiety. In addition, Brooks et al. (2006) found a second‐order factor structure of the K10 among Austrian adults. The four first‐order factors included negative affect, fatigue, nervousness, and agitation. As for the second‐order factors, negative affect and fatigue loaded on depression and nervousness and agitation loaded on anxiety. This second‐order factor structure was also reported in Zhou et al.’s study (2008) among Chinese college students. Figures of these alternative factor structures are provided in the Supporting Information of this study.

The issue of measurement invariance (MI) has been discussed by many researchers over the years (e.g., Brown, 2015; Byrne & Watkins, 2003; Meredith, 1993; Vandenberg & Lance, 2000). In general, a measure that has MI indicates that it measures the same construct across groups (such as different races, genders, ages, schools). It implies that the interpretation of the measure is the same across groups. MI of the K10 across different groups indicates that the K10 measures psychological distress comparably across groups. To establish that its measurement properties are equivalent across male and female respondents, for example, researchers need to ensure that a given score on the K10 represents the true level of psychological distress in male and female respondents. Unfortunately, we did not find any studies that have systematically tested cross‐gender invariance of the K10.

However, we found one study that examined the MI of the K6 (an abbreviated version of the K10) among young, middle‐aged, and older adults from Canada (Drapeau et al., 2010). They reported full MI across gender among middle‐aged adults and partial MI across gender among the other two age groups (Drapeau et al., 2010). Another study based in Canada demonstrated K6's MI between youth and adults and between male and female youth (Ferro, 2019). Conversely, researchers have identified measurement noninvariance across gender in the K6 item thresholds in a large sample of Australian adolescents, indicating that reporting biases may be present in the K6 items (Mewton et al., 2016). We also found some evidence on the MI across gender using different measures. One study, for example, reported full MI across gender among Chinese college students using the Depression Anxiety Stress Scales‐21 (Lu et al., 2018).

Based on our literature review, we identified a few gaps to be filled in this study. First, the evidence on the factor structure of the K10 is largely inconsistent. Thus, there is a need to compare these structures for better validation of the K10. Second, based on our review, studies on the K10's MI among adolescents are not available. Therefore, the role of gender biases using the K10 to assess psychological distress in adolescence remains unknown. These gaps undoubtedly confound the findings of subgroup differences in psychological distress among adolescents. It is unclear whether observed differences are the result of true differences in psychological distress across genders, due to gender biases in the measurement instrument, or because of some combination of both. To bridge these gaps, this study aimed to identify the best‐fitting factor structure of the K10 and investigate whether this structure holds at different levels of MI among a group of migrant children in Hangzhou, China.

## 2 METHOD

### 2.1 Sample procedures

Using convenience sampling, we selected a sample in a private middle school in Hangzhou, China. As one of the largest metropolitan areas in China (with almost 10 million residents in 2018), this city attracts many migrant workers from different parts of China. Many children migrate with their parents to Hangzhou and go to elementary and middle schools in the city. Due to the household registration system (*hukou*), a large number of migrant children without a local urban hukou status in Hangzhou are excluded from urban public schools. Even though some of them attend public schools, more go to private schools that are designated to enroll only children of migrants. Comparing to public schools, these schools are often not adequately funded. We selected a private middle school that enrolled only children of migrants in a region of the city, where migrants and their families tended to congregate. After being introduced to the principal of the school by a local government agency that oversees the school, we met with the principal and explained the purpose of our study. The principal agreed to support our study by helping us recruit teachers of grades 6 to 8, who then administered the study questionnaire in their class. The study was approved by the first author's university human research ethics committee (Approval #: 2018415).

We distributed the parent consent forms through six teachers who agreed to administer the questionnaire in their classes. Students were instructed by their teachers to bring the forms back after their parents signed the consent form for their children to participate in the study. A total of 339 students were able to bring the form back before our scheduled date for the data collection. Among the 339 parents who signed the consent form, all agreed to have their children participate in our study. On the day when we collected data in six different classrooms, students were asked to assent to participate before they filled in a paper‐and‐pencil survey. They were told that their participation in the study was completely voluntary, and they may withdraw at any time. All students assented to participate. In addition to the demographic information, the questionnaire included questions on their psychological stress, school environment, and family relationships.

Our initial sample size was 339. We excluded observations with missing values on gender. The size of the analytic sample of our study was 327. It has been proposed to use the ratio of the number of participants to the number of variables to determine whether the sample size is adequate (Kline, 1994). Considering previous researchers have recommended a ratio of 20 to 1 as the cutoff (Hair et al., 1998), we determined that our analytic sample size was more than adequate, with the ratio equal to 32.1 to 1.

### 2.2 Measure

This present study focused on psychological distress. In our survey, it was measured by the Chinese version of the K10 (Bu et al., 2017), which was translated from the original K10 developed by Kessler et al. (2002) in the United States. The questionnaire starts with a prompt: “These questions concern how you have been feeling over the past 30 days. Tick a box below each question that best represents how you have been.” Sample questions include “During the last 30 days, about how often did you feel tired out for no good reason?” and “During the last 30 days, about how often did you feel hopeless?” All items are answered on a 5‐point Likert type scale: 0 = none of the time; 1 = a little of the time; 2 = some of the time; 3 = most of the time; 4 = all of the time.

## 3 DATA ANALYSIS STRATEGIES

All analyses were conducted using Mplus version 8.3 (Muthén & Muthén, 2019). Data and Mplus program code can be requested from the authors. Weighted least‐squares means and variance adjusted (WLSMV) estimator was used to estimate all CFA models because our data were non‐normal with categorical nature: the Henze‐Zirkler multivariate normality test statistic = 30.98, *p *= < .001. One of the WLSMV's requirements is that all indicators must have the same number of categories. When the data on the variables of the same scale do not have the same number of categories, researchers have recommended collapsing the adjacent response categories (Liu et al., 2017). Since our participants' ratings of some K10 items did not cover all five categories, we collapsed the responses into three categories by combining "a little of the time" with "some of the time" and "most of the time" with "all of the time." Also, theta parameterization was specified to allow for the residual variances of the factor indicators as parameters.

To identify a best‐fitting model, we used CFA to compare alternative factor structures of the K10. To compare factor structures, we used the model proposed by Brooks et al. (2006) as the baseline, in which the K10 included four first‐order factors and two second‐order factors. Nested in the baseline model, two additional factor structures indicated in previous research were tested subsequently: a two‐factor model (Bu et al., 2017) and a one‐factor model (Kessler et al., 2002).

Subsequently, we used CFA for ordered‐categorical measures (CFA‐OCM) to test K10's cross‐gender MI and structural invariance. Following recommendations in the literature (Brown, 2015), we conducted the analyses in the following order: (1) separate CFA in each group; (2) CFA with equal form (configural invariance); (3) CFA with equal factor loadings (metric or weak invariance); (4) CFA with equal indicator intercepts (scalar or strong invariance); (5) CFA with equal indicator residual variances (strict invariance). In Mplus, categorical indicator thresholds are modeled instead of intercepts, with the number of thresholds equal to the number of categories minus one (Muthén & Muthén, 2017). We also tested population heterogeneity by constraining factor variances, covariances, and means to be equal.

Measures of model fit included the *χ* ^2^ goodness of fit, the root‐mean‐square error of approximation (RMSEA), comparative fit index (CFI), and the Tucker–Lewis index (TLI). Nonsignificant *χ* ^2^ goodness of fit was used to define sufficient model fit. Also, as recommended in previous research (Brown, 2015; Hu & Bentler, 1999), a RMSEA value close to 0.06 or below, an SRMR value close to 0.08 or below, and CFI and TLI values close to 0.95 or greater were used to define good fit. Finally, to compare model fit across nested models, the DIFFTEST option was used to calculate *χ* ^2^ differences in all Mplus models.

Many K10 items had one missing observation (items 1, 4, 7, 8, 9, and 10) and item 3 had two missing observations. Missingness on K10 was handled by the WLSMV estimator using pairwise deletion (WLSMV‐PD) in Mplus. Research has shown that WLSMV‐PD produces unbiased model estimates and is far more efficient than the WLSMV estimator using listwise deletion (Asparouhov & Muthén, 2010). Though this method has been criticized for inflating type I error, this inflation is only prominent when the rate of missing data is high (Chen et al., 2020).

## 4 RESULTS

The mean age of the total sample was 13.39 years (SD = 1.02; range = 11–16). There was no significant difference in age between male and female adolescents (*p* = .58). Students were almost equally distributed across grade levels: 36% were sixth graders, 33% seventh graders, and 31% eighth graders. No difference was detected in the bivariate relationship between gender and grade levels: *χ* ^2^(2) = 0.48, *p* = .79. We conducted *t*‐tests to examine the differences in K10 item scores across gender. As shown in Table 1, female adolescents were more likely to feel nervous (*t* = 2.21, *p* = .01), hopeless (*t* = 1.84, *p* = .03), depressed (*t* = 2.54, *p* = .006), severely depressed (*t* = 1.91, *p* = .03), and worthless (*t* = 1.71, *p* = .04) than their male counterparts. However, there were no differences in students’ report on the other five items: tired, extreme nervous, restless, extreme restless, and effort.

**TABLE 1:** Descriptive statistics and *t*‐tests of the K10 items by gender

		*n* ^a^		Skewness		Kurtosis		*M* (SD)			
#	Item	Female	Male	Female	Male	Female	Male	Female	Male	*t* ^b^	*p*
1	Tired	135	191	2.09	2.36	6.17	7.96	1.26 (0.57)	1.17 (0.42)	1.58	.06
2	Nervous	135	192	1.97	3.22	6.12	13.31	1.22 (0.47)	1.12 (0.37)	2.21	.01
3	Severely nervous	134	191	3.02	4.62	11.88	25.49	1.11 (0.34)	1.07 (0.29)	1.25	.11
4	Hopeless	135	191	2.59	3.88	8.93	18.01	1.19 (0.48)	1.10 (0.36)	1.84	.03
5	Restless	135	192	2.19	2.79	6.99	10.41	1.22 (0.50)	1.15 (0.41)	1.41	.08
6	Severely restless	135	192	3.39	4.90	14.53	27.55	1.11 (0.36)	1.07 (0.31)	1.17	.12
7	Depressed	134	192	2.57	4.40	8.97	23.29	1.18 (0.46)	1.07 (0.30)	2.54	.006
8	Effort	135	191	2.85	3.44	10.14	14.24	1.17 (0.48)	1.13 (0.42)	0.89	.19
9	Severely depressed	135	191	2.34	3.48	7.68	14.96	1.21 (0.49)	1.12 (0.38)	1.91	.03
10	Worthless	134	192	2.83	3.89	10.05	18.40	1.17 (0.48)	1.09 (0.34)	1.71	.04

^a^ The total number of observations for each group varied because of missing data on all items except items 2, 5, and 6.

^b^ We used *t*‐tests because all K10 item scores were originally rated on a scale of 1–5 and recoded on a scale of 1–3.

### 4.1 CFA model comparison

To determine the best‐fitting model, we compared three CFA models: the second‐order model identified by Brooks et al. (2006), the two‐factor model validated by Bu et al. (2017), and the unidimensional one‐factor model reported by Kessler et al. (2002). As shown in Table 2, all three models fit the data well. The two‐factor model did not significantly worsen the fit of the second‐order model (*D_χ_* ^2 ^= 1.60, *df* = 4, *p* = .81). The unidimensional model, on the other hand, significantly worsened the fit of the two‐factor model (*D_χ_* ^2 ^= 8.10, *df* = 1, *p* = .004). We decided to retain the two‐factor model for further analyses because it was the best‐fitting model with the least number of parameters. This model is also consistent with substantive theory, which suggests that common anxiety symptoms include agitation and nervousness and common depressive symptoms include negative affect. Previous research has indicated that fatigue is associated with both anxiety and depression disorders (Sharpe & Wilks, 2002). Our model captured fatigue on both anxiety and depression, with the item of tired loading on anxiety and the item of effort loading on depression.

**TABLE 2:** CFA models comparing factor structures

	*χ* ^2^	*df*	*p*	CFI	TLI	RMSEA [90% CI]	SRMR
Second‐order model	40.99	30	.09	0.994	0.991	0.033 [0.000, 0.057]	0.046
Two‐factor model	42.23	34	.16	0.996	0.994	0.027 [0.000, 0.051]	0.048
One‐factor model	56.82	35	.01	0.988	0.985	0.044 [0.021, 0.064]	0.059

### 4.2 Separate baseline models

Our baseline model was the two‐factor model retained after comparing alternative CFA models. The baseline model was tested in boys and girls separately, while factor variances and factor means were fixed to 1 and 0, respectively. Results indicated a well‐fitting model for both boys (*χ* ^2^ = 36.21, *df* = 34, *p* = .37; CFI = 0.998; TLI = 0.998; RMSEA = 0.018, 90% CI = [0.000, 0.057]; SRMR = 0.062) and girls (*χ* ^2^ = 32.12, *df* = 34, *p* = .56; CFI = 1.000; TLI = 1.000; RMSEA = 0.000, 90% CI = [0.000, 0.058]; SRMR = 0.061). For boys, standardized factor loadings ranged from 0.78 to 0.94, all significant at the *p* < .001 level. For girls, standardized factor loadings ranged from 0.74 to 0.88, all significant at the *p* < .001 level.

### 4.3 Measurement invariance

To examine cross‐gender MI, we first tested the baseline model in both gender groups concurrently (Model 1). This equal form model is also referred to as the configural invariance model in the literature. The factor variances were fixed to 1 and the factor means were fixed to 0 in both groups. The residual variances were all constrained to 1 in both groups. All item factor loadings (one per item) and thresholds (two per item given three response options) were estimated. This model showed excellent fit (Table 3), suggesting that configural invariance was supported.

**TABLE 3:** Model fit of measurement invariance models

	*χ* ^2^	*df*	*p*	Δ*χ* ^2^	Δ*df*	CFI	TLI	RMSEA [90% CI]	SRMR
Model 1: Equal form	68.51	68	.46			1.000	1.000	0.007 [0.000, 0.047]	0.062
Model 2: Equal factor loadings	76.04	76	.48	7.72	8	1.000	1.000	0.002 [0.000, 0.045]	0.072
Model 3: Equal indicator thresholds	91.90	94	.54	14.66	18	1.000	1.000	0.000 [0.000, 0.040]	0.067
Model 4A: Equal residual variances	82.05	84	.54			1.000	1.000	0.000 [0.000, 0.041]	0.064
Model 4B: Equal residual variances	91.90	94	.54	9.40	10	1.000	1.000	0.000 [0.000, 0.040]	0.067

**FIGURE 1:**
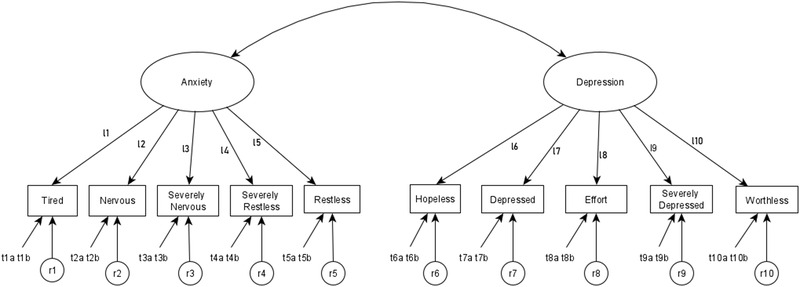
Model parameters constrained in testing the MI of the K10. *Note*. Parameters l1–l10 stand for factor loadings, which were constrained to be equal across the two groups in Model 2 (metric invariance); t1a and t1b through t10a and t10b stand for item thresholds (two thresholds for each item), which were constrained to be equal across groups in Model 3 (scalar invariance); r1–r10 stand for item residual variances, which were fixed to 1 for both groups in Model 4 (strict invariance)

In subsequent models, we proceeded by applying more stringent parameter constraints to examine potential decreases in fit resulting from measurement or structural noninvariance between boys and girls, with boys as the reference group. Figure 1 explains how we constrained different parameters in four steps (i.e., models). In addition, model comparison statistics, as well as model fit information of all these models, are presented inTable 3.

Equality of the unstandardized item factor loadings between groups was examined in a metric invariance model (Model 2). The factor variances were fixed to 1 in boys for identification but were freely estimated in girls; the factor means were fixed to 0 in both groups for identification. All factor loadings were constrained equal across groups, all item thresholds were estimated, and all residual variances were constrained to 1 across groups. Model 2 did not fit significantly worse than Model 1: DIFFTEST (8) = 7.72, *p *= .46. Metric invariance was thus supported, indicating that the same latent factors (i.e., depression and anxiety) were being measured in each gender group by the K10.

Equality of the unstandardized item thresholds across groups was examined in a scalar invariance model (Model 3). The factor variances and means were fixed to 1 and 0, respectively, in boys for identification, but they were freely estimated for girls. All factor loadings and item thresholds were constrained equal across groups; all residual variances were constrained equal to 1 in both groups. Model 3 did not significantly worsen the fit of Model 2: DIFFTEST (18) = 14.66, *p *= .69. Thus, scalar invariance was supported, suggesting that the observed difference in the proportion of responses in each category for all K10 items was due to factor mean differences only.

Next, we tested the invariance of the unstandardized residual variances across groups (i.e., strict invariance). The model comparison at this step proceeded backward. In other words, a model (Model 4A) with all residual variances freely estimated in girls was estimated first. It was then compared with a model in which all residual variances were fixed to 1 in girls (Model 4B). The residual variances in the boys were all fixed to 1 for identification in both models, and the rest of the model parameters were estimated as described in Model 3. Model 4B did not fit significantly worse than the Model 4A: DIFFTEST (10) = 9.40, *p *= .49. Thus, strict invariance was supported, indicating that the amount of item variance not accounted for by the two factors was the same in all K10 items across groups.

### 4.4 Structural invariance

**FIGURE 2:**
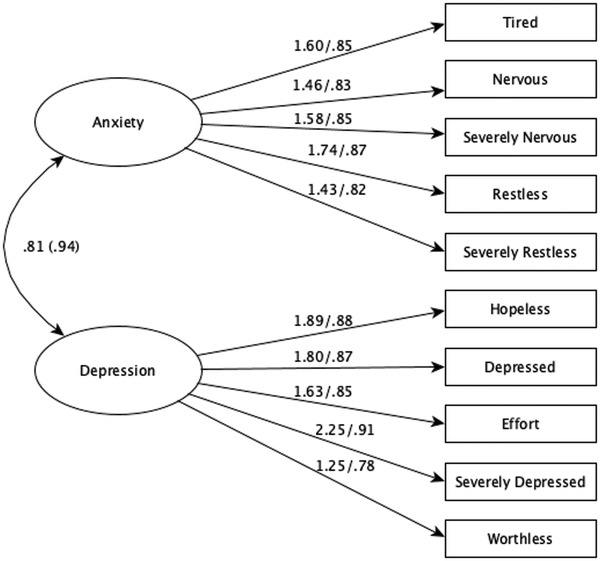
K10 factor loadings based on full MI and partial structural invariance. *Note*. All loadings were the same for boys and girls and significant at the *p* < .001 level; coefficient before slash is unstandardized and coefficient after slash is standardized. Factor covariance outside of parentheses was for girls and factor covariance inside parentheses was for boys. Cronbach's α = .80 for anxiety and .82 for depression. *χ* ^2^ = 99.61, *df* = = 96, *p* = .38; CFI = 0.998; TLI = 0.998; RMSEA = 0.015, 90% CI = [0.000, 0.045]; SRMR = 0.073

Based on the strict invariance model (i.e., Model 4B), we tested an additional model for structural invariance. In this new model (Model 5), we first tested the invariance in the two‐factor variances by fixing them to 1 in both groups. This resulted in a nonsignificant decrease in model fit relative to Model 4B: DIFFTEST (2) = 4.09, *p *= .13, indicating that the invariance in factor variances was achieved in our sample. Model 5 yielded excellent model fit: *χ* ^2^ = 99.61, *df* = 96, *p* = .38; CFI = 0.998; TLI = 0.998; RMSEA = 0.015, 90% CI = [0.000, 0.045]; SRMR = 0.073. Model parameters for Model 5 are given in Figure 2. As shown inFigure 2, standardized factor loadings ranged from 0.82 to 0.87 for anxiety and from 0.78 to 0.91 for depression.

Then we used the MODEL TEST command in Mplus to probe the invariance in the factor covariance. Model specifications remained the same as Model 5. Results indicated that the covariance between depression and anxiety was not significantly different across groups: Wald statistic = 1.39, *df* = 1, *p* = .24. The factor covariance was 0.81 (*p* < .001) and 0.94 (*p* < .001) for girls and boys, respectively. Finally, we examined the difference in factor means across genders based on Model 5: the mean of depression for girls was significantly different from 0 (*M* = 0.37, SE = 0.17, *p *= .03), and the mean of anxiety for girls was also significantly different from 0 (*M* = 0.32, SE = 0.16, *p *= .05). These results indicated that, on average, girls experienced higher levels of depression and anxiety than boys, whose factor means were 0. We also used *t*‐tests to examine the mean difference in anxiety and depression between girls and boys based on the composite score of each subscale. For anxiety, the mean difference was 0.07 (*t* = 2.07, *p* = .04, Cohen's *d* = 0.23); for depression, the difference was 0.08 (*t* = 2.28, *p* = .02, Cohen's *d* = 0.26). Thus, it was corroborated that girls displayed higher levels of psychological distress than boys, though the effect size was relatively small.

## 5 DISCUSSION

Based on a convenience sample of children of Chinese internal migrants living in Hangzhou, China, this study's aim is twofold: (1) to identify the best‐fitting factor structure of the K1, and (2) to examine the cross‐gender MI of the K10 using the best‐fitting model. Our findings indicated that the two‐factor model validated by Bu et al. (2017) fit our data best, which was retained for further data analysis in our study. This best‐fitting model includes anxiety and depression as two factors, with five items loading on each factor. Specifically, depression includes items of hopeless, depressed, effort, severely depressed, and worthless, whereas anxiety includes items of tired, nervous, severely nervous, restless, and severely restless. Different from O'Connor et al.’s two‐factor model (2012), the item of tired loaded on anxiety rather than depression in our model. Additionally, different from Sunderland et al.’s two‐factor model (2012), the item of effort loaded on depression rather than anxiety in our model.

In terms of the testing of MI for the K10, our analyses showed that full MI of the K10 was obtained across boys and girls, that is, the relationships of the K10 items to the latent factors of depression and anxiety were equivalent between boys and girls. Specifically, boys and girls in our sample appear to use the same conceptual framework, as assessed with the K10, in their perception of psychological distress (equal form); the items of the K10 scale have similar meanings for boys and girls (equal factor loadings); the scaling of the K10 items is similar for the compared groups in our sample (equal item thresholds); the K10 items demonstrate similar amounts of unique variance across boys and girls (equal item residual variances). However, structural invariance was not obtained in full, such that girls experienced higher levels of depression and anxiety than boys. Taken together, our findings suggested that gender differences in anxiety and depression among Chinese migrant children, as measured by the K10, can be construed as meaningful and true, and not due to measurement error or gender biases associated with the use of the K10.

Our findings of invariance are encouraging because MI is a precursor to any subgroup comparison using the K10. Our study also highlights the necessity of establishing MI when comparing subgroup differences. For future researchers, it is always prudent to examine MI before conducting subgroup analyses. This is an imperative lesson considering that ample research has studied gender differences in anxiety and depression symptomatologies but not much has established MI as a precondition (e.g., Lai, 2011; Parker et al., 2014; Schuch et al., 2014; Silverstein et al., 2013). Given our findings of invariance, our study's findings support the validity of these comparative studies. However, this is true only to some extent in that the measurement in our study is not necessarily the same as used in these comparative studies.

Although previous research on the MI of psychological distress is rare, existing evidence reveals MI of depressive symptoms (as measured by Children's Depression Inventory) across male and female adolescents (Carle et al., 2008). Also, a few studies have provided evidence on the invariance (or partial invariance) of the K6 (a shorter version of the K10) across genders and different age groups (Drapeau et al., 2010; Ferro, 2019; Mewton et al., 2016). To better illuminate the role of gender biases in the measurement of psychological distress, future researchers may need to consider testing the MI of the K10 across age groups (especially between children and adults). In addition, previous research (e.g., Bender et al., 2012; Derdikman‐Eiron et al., 2011; Negriff & Susman, 2011) has repeatedly reported that girls experience higher levels of psychological distress than boys. Due to the higher means of anxiety and depression among girls, our study confirms this observation.

Both factors validated in our study are common mental illnesses familiar to mental health clinicians. Five K10 items fit the common characterization of anxiety (including two items of agitation, two items of nervousness, and one item of fatigue) and the other five items fit that of depression (including four items of negative affect and one item of fatigue). Probably due to the inclusion of these commonly understood mental health symptomatologies, K10 has been widely used by mental health professionals as a screening tool for common mental health problems in both practice and research (e.g., Cornelius et al., 2013; Furukawa et al., 2003; Kessler, Berglund, et al., 2003; Thelin et al., 2017). Berle et al. (2010) have argued that it makes more sense to understand the K10 as a specific measure of anxiety and depression, despite Kessler et al.’s (2002) intent to use the K10 to measure “nonspecific” psychological distress. They contended that a complete model of psychological distress should encompass other aspects such as somatic complaints and even psychotic symptoms, which are not assessed with the K10 (Berle et al., 2010). However, this argument does not preclude the use of the K10 as an effective tool for common mental health problems such as anxiety and depression.

By demonstrating MI of the K10, the present study supports the notion that girls experience higher levels of anxiety and depression than boys. More importantly, the differences are not due to measurement variability, thus reflecting the true differences between genders. Logically, the next step in mental health practice involves eliminating gender disparities by reducing anxiety and depression symptomatologies among girls. Interventions are often closely monitored to achieve the best outcomes with clients. Our findings indicate that the K10 could be used to evaluate the effectiveness of such interventions.

### 5.1 Implications for practice

In mainland China, where school‐based mental health interventions are not always readily available, it is more practical to consider using the K10 as a screening tool among school‐aged children. The brevity and validity of the K10 lend itself to being widely used as a mental health assessment tool regularly. We believe that middle school students are worthy of more attention compared to other school‐aged children because research has shown that mental health problems developed in childhood can amplify in early adolescence or the middle school years (Stormshak et al., 2011) and that gender differences in depression symptomatologies often emerge reliably around age 15 (Carle et al., 2008). For Chinese mental health professionals, it may be more practical to assess students with migrant parents, considering their relatively vulnerable status. Based on our findings, female children of migrants are at a higher risk than their male counterparts. Therefore, the assessment with female students should also be prioritized.

### 5.2 Limitations and strengths

The current study has several limitations. First, our sample was limited geographically to a middle school for children of urban‐to‐rural migrants in Hangzhou, China, thus our findings cannot be generated to all migrant children in Hangzhou or those in other parts of China. Future studies should attempt to address this limitation by including a nationally representative sample of migrant children. Second, our study focused on middle school students who migrated with their parents to cities. A different group of migrant children, labeled as “left‐behind” children, who do not migrate with their parents and constitute roughly two‐thirds of all migrant children in China (Tong et al., 2019), were not included in our study. We also left out nonmigrant children who were born and lived with their parents in rural or urban areas. Future research could tap into the experiences of psychological distress among these populations and examine MI of the K10 across left‐behind children and non‐left‐behind children and across migrant and nonmigrant children. Third, our study did not test gender differences in K10's criterion validity (e.g., its ability to predict some psychiatric disorders). Fourth, the current study was based on the Classic Theory Test (CTT) and future researchers may wish to examine K10's invariance based on the Item Response Theory (IRT), which can provide additional information, such as item difficulty, discriminative ability, and differential item functions (Ye et al., 2018).

Nevertheless, this study adds to the K10 literature in several ways. It provides a confirmatory test of the factor structure of the K10 and identifies a best‐fitting factor structure among a sample of middle school students. By conducting MI analyses, it explicitly examines whether the subgroup difference in psychological distress, as assessed with the K10, is a result of measurement bias. Finally, it uses a community sample of children of rural‐to‐urban migrants in China, who are considered as a vulnerable population, not only due to their lower socioeconomic status but also due to the discrimination associated with their migrant status. These conditions may predispose migrant children to experience higher levels of psychological distress. Based on our results, it is our sincere hope that Chinese schools with migrant children will provide more screening services using the K10.

## 6 CONCLUSIONS

This study confirms that the two‐factor structure (five items on depression and five on anxiety) is the best‐fitting model for the K10 among Chinese migrant children. It also supports the full MI across gender, thus eliminating the possibility of measurement error and gender biases associated with the use of the K10 among this population. These findings provide important implications for future research and practice.

### PEER REVIEW

The peer review history for this article is available at https://publons.com/publon/10.1002/brb3.2417

## CONFLICT OF INTEREST

The authors declare no conflict of interest.

## Supporting information

Supporting Information

## Data Availability

The data that support the findings of this study are available from the corresponding author upon reasonable request.
