# Supplementary material for: Measurement invariance of the Kessler Psychological Distress Scale (K10) among children of Chinese rural‐to‐urban migrant workers
Source: Brain Behav. 2021 Nov 13;11(12):e2417. doi: 10.1002/brb3.2417 (PMC8671765; doi:10.1002/brb3.2417)

**Supplemental Figure 1**

*Unidimensional Factor Model of the K10 as Validated by Kessler and Colleagues (2002)*


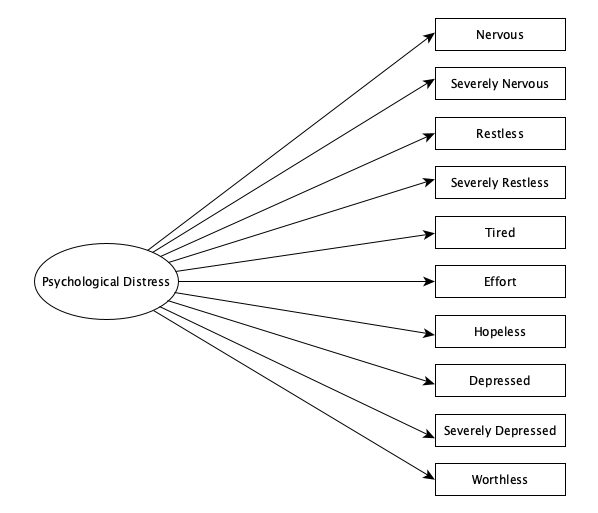


**Supplemental Figure 2**

*Two-Factor Model of the K10 as Validated by Bu and Colleagues (2017)*


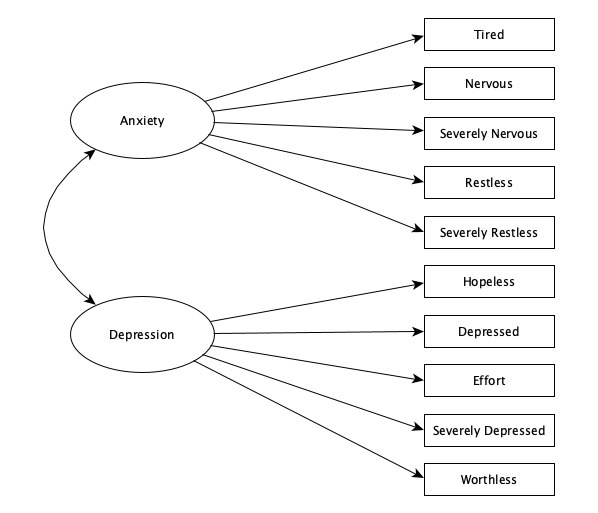


**Supplemental Figure 3**

*Second-Order Factor Model of the K10 as Validated by Brooks and Colleagues (2006)*


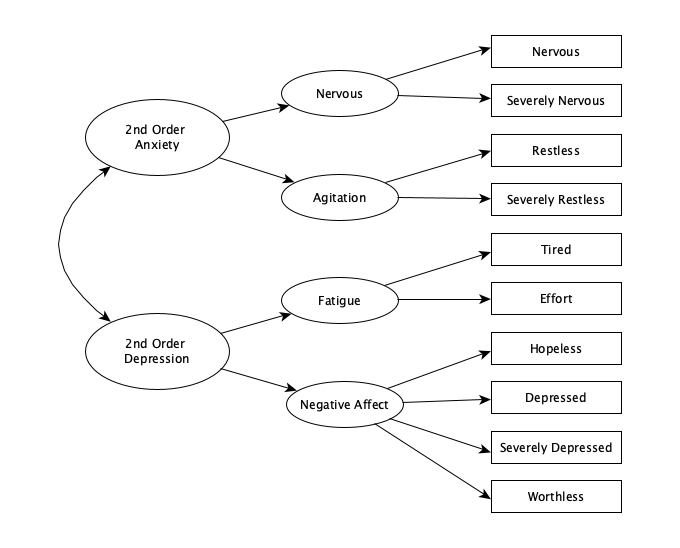

Supplement: Supplementary file 1 — Supporting Information [file BRB3-11-e2417-s001.docx]
